# Localization and potential role of prostate microbiota

**DOI:** 10.3389/fcimb.2022.1048319

**Published:** 2022-12-07

**Authors:** Koichi Okada, Kentaro Takezawa, Go Tsujimura, Takahiro Imanaka, Sohei Kuribayashi, Norichika Ueda, Koji Hatano, Shinichiro Fukuhara, Hiroshi Kiuchi, Kazutoshi Fujita, Daisuke Motooka, Shota Nakamura, Yoshihisa Koyama, Shoichi Shimada, Norio Nonomura

**Affiliations:** ^1^ Department of Urology, Osaka University of Graduate School of Medicine, Suita, Japan; ^2^ Department of Urology, Faculty of Medicine, Kindai University Hospital, Osakasayama, Japan; ^3^ Department of Infection Metagenomics, Genome Information Research Center, Osaka University Research Institute for Microbial Diseases, Suita, Japan; ^4^ Department of Neuroscience and Cell Biology, Osaka University Graduate School of Medicine, Suita, Japan

**Keywords:** prostate, microbiota, microbiome, benign prostate enlargement, beneign prostate hyperplasia, *in situ* hybridization

## Abstract

**Introduction:**

We aimed to clarify the presence and localization of the prostate microbiota and examine its association with benign prostate enlargement (BPE).

**Methods:**

The microbiota of prostate tissues and catheterized urine from 15 patients were analyzed by 16S metagenomic analysis and compared to show that the prostate microbiota was not a contaminant of the urinary microbiota. Fluorescence in situ hybridization (FISH) and in situ hybridization (ISH) using the specific probe for eubacteria was performed on prostate tissue to show the localization of bacteria in the prostate. The BPE group was defined as prostate volume ≥30 mL, and the non-BPE group as prostate volume <30 mL. The microbiota of the two groups were compared to clarify the association between prostate microbiota and BPE.

**Results:**

Faith’s phylogenetic diversity index of prostate tissue was significantly higher than that of urine (42.3±3.8 vs 25.5±5.6, P=0.01). Principal coordinate analysis showed a significant difference between the microbiota of prostate tissue and catheterized urine (P<0.01). FISH and ISH showed the presence of bacteria in the prostatic duct. Comparison of prostate microbiota between the BPE and non-BPE groups showed that the Chao1 index of the BPE group was significantly lower than that of the latter [142 (50–316) vs 169 (97–665), P=0.047] and the abundance of Burkholderia was significantly higher in the BPE group than in the latter.

**Conclusions:**

We demonstrated that the prostate microbiota was located in the prostatic duct and reduced diversity of prostate microbiota was associated with BPE, suggesting that prostate microbiota plays a role in BPE.

## Introduction

Benign prostate enlargement (BPE) is caused by benign prostate hyperplasia (BPH) and may cause bladder outlet obstruction, leading to lower urinary tract symptoms (LUTS) such as decreased urinary flow and increased urinary frequency (Lerner et al., 2021). LUTS associated with BPE (LUTS/BPE) is a common disease that progresses with aging, affecting >50% of men aged >50 years and increasing to 80% of men aged >80 years (Egan, 2016). Treatment of LUTS/BPE is primarily medical, including alpha-blockers, 5-α reductase inhibitors, and phosphodiesterase 5 selective inhibitors. Welliver et al. reported that medication use increased with time from 2004 to 2013 in all ages (Welliver et al., 2020). However, LUTS/BPE is a progressive disease (Emberton et al., 2008). The CombAT study reported that clinical progression of LUTS and BPH over 4 years was seen in 21.5% of patients treated with tamsulosin and 12.6% of patients treated with tamsulosin and 5-α reductase inhibitors (Roehrborn et al., 2010). One of the reasons why it is difficult to prevent progression of LUTS/BPE is that the mechanism of BPH has not been clarified. It is reported that aging, androgens, estrogen, growth factors, and chronic inflammation are associated with BPE (Devlin et al., 2021). Recently, we have reported that gut microbiota is also associated with BPE (Takezawa et al., 2021). However, although some factors have been identified, the exact etiology of LUTS/BPE is unknown (Vuichoud and Loughlin, 2015). Therefore, there must be new factors associated with BPH and BPE. The discovery of new factors associated with BPE would allow new approaches to prevent and treat LUTS/BPE.

Next-generation sequencing has made it possible to study microbiota without culture and has revealed the existence of the urinary microbiota (Wolfe et al., 2012; Hilt et al., 2014). The urinary microbiota has been reported to be associated with LUTS, including overactive bladder, interstitial cystitis, chronic prostatitis, and chronic pelvic pain syndrome (Antunes-Lopes et al., 2020). These associations suggest that local microbiota play some role in the development of disease. Recently, the presence of the prostate microbiota was suggested. Jain et al. performed 16S metagenomic analysis of prostate tissue obtained by transurethral resection from 20 patients and detected 1239 bacterial species (Jain et al., 2020). However, there is concern about contamination from the urinary microbiota in collecting prostate tissue. Therefore, a more detailed examination taking contamination into consideration is needed in studying the presence of the prostate microbiota. Given the prostate microbiota exists, its location and function are unknown. In the current study, we aimed to confirm the presence of the prostate microbiota, and investigated its location and relationship with BPE.

## Materials and methods

### Comparisons of microbiota in prostate tissue and catheterized urine

In investigating the presence of prostate microbiota, there is concern that the microbiota of prostate tissue does not reflect prostate microbiota but is just a contaminant of the urinary microbiota. Therefore, we compared the microbiota of prostate tissue with that of catheterized urine, which reflects the urinary microbiota. We collected both prostate tissue and catheterized urine from patients who were admitted for surgery including robot assisted laparoscopic prostatectomy (RALP), holmium laser enucleation of prostate (HoLEP), and subcapsular prostatectomy between January 2020 and July 2021 at Osaka University Hospital. Prostate tissue samples were collected aseptically. Briefly, at the time of RALP, we took a needle biopsy from the excised prostate specimen using a 16-gauge side-notch needle (PRIMECUT II, Boston Scientific, Marlborough, MA, United States), aiming at the transition zone that was estimated to be cancer-free on preoperative magnetic resonance images. At the time of HoLEP, we obtained a resection fragment of the enucleated prostate tissue with dry heat-sterilized forceps. One of the samples was immediately frozen and stored at −80 °C until bacterial DNA extraction. The other specimens were used to make formalin-fixed, paraffin-embedded (FFPE) blocks. When prostate cancer was detected by hematoxylin and eosin (HE) staining of FFPE slices, the case was excluded. Catheterized urine samples of up to 50 mL were collected in the operating room prior to surgery. The urine samples were centrifuged at 9200 g for 20 min at 4 °C and the supernatants were decanted, leaving 2 mL. The centrifuged urine samples were vortexed and stored at −80 °C until all the samples were collected.

After all prostate tissue and urine specimens were collected, they were submitted to bacterial DNA extraction and analysis of microbiota as reported by Kameoka et al. (Kameoka et al., 2021). The bacterial DNA was extracted from the samples using DN easy Power Soil Kit (Qiagen, Venlo, The Netherlands). Amplicons targeting the V1–V2 variable regions of the 16S rRNA gene were generated using the primers 27Fmod (5'-AGRGTTTGATCMTGGCTCAG-3') and 338R (5'-TGCTGCCTCCCGTAGGAGT-3'). Then, a 251-bp paired-end sequencing of the amplicons was performed using a MiSeq (Illumina, San Diego, CA, USA). The paired-end sequences obtained were merged, filtered, and denoised using DADA2 (Callahan et al., 2020). Taxonomic assignment was performed using QIIME2 feature-classifier plugin with the Greengenes 13_8 database. The QIIME2 pipeline, version 2020.2, was used as the bioinformatics environment for the processing of all relevant raw sequencing data.Alpha diversity was evaluated by rarefaction analysis of the Chao1 index, Faith’s phylogenetic diversity (PD) index, and Shannon index. The values in 5368 sequences were statistically compared. Comparisons between groups were made using the paired-samples *t*-test. Beta diversity was assessed *via* principal coordinate analysis (PCoA) based on unweighted UniFrac distance and analysis of similarities. *P*<0.05 was considered significant. PCoA was performed in QIIME II, and other statistical tests were performed using JMP Pro 16 (SAS Institute Inc., Cary, NC, USA). Linear discriminant analysis effect size (LEfSe) was performed using the Galaxy web application (https://huttenhower.sph.harvard.edu/galaxy/ ) to identify significant differences in operational taxonomic units (OTUs) between the prostate tissue and urine specimen. The threshold on the logarithmic LDA score for discriminative features was 2.0.

### Fluorescence *in situ* hybridization for eubacteria in prostate tissue

Fluorescence *in situ* hybridization (FISH) for eubacteria was performed to histologically examine the presence and localization of prostate microbiota. FISH was carried out on mouse intestine FFPE slices to test the performance of the specific probe for eubacteria (EUB338 probe, 5'-GCTGCCTCCCGTAGGAGT-3') (Amann et al., 1990) and the negative probe for eubacteria (NON338, 5'-ACATCCTACGGGAGGC-3') (Wallner et al., 1993) labeled with cyanine 5. FISH was performed on 20 μm-thick serial sections of frozen prostate tissue that were embedded in Tissue-Tek™ O.C.T compound (Sakura Finetek, Torrance, CA, USA). The slides were fixed in 10% formalin after being sliced. Prior to FISH, the sections were treated with TrueBlack (Biotium Inc. Hayward, CA, USA) to reduce nonspecific fluorescence. Hybridization was conducted as described previously (Hsu et al., 2021). Sections were incubated overnight at 40 °C with EUB338 probe or NON338. The sections were rinsed and mounted with VECTASHIELD HardSet Antifade Mounting Medium with 4',6-diamidino-2-phenylindole (Vector Laboratories) and analyzed using a microscope (BZ-X710, Keyence, Osaka, Japan).

### ISH for eubacteria in prostate tissue

ISH for eubacteria in prostate tissue was performed to examine detailed localization of the prostate microbiota. Gram stain and ISH were performed to establish that the cells detected in ISH were bacteria. The sections used for ISH were the same as for FISH. The sections were incubated and denatured as reported previously (Ikeda et al., 2011). Hybridization and rinsing were carried out with EUB338 or NON338 conjugated with digoxigenin under the same conditions as for FISH. Peroxidase-blocking solution (Dako, Glostrup, Denmark), 5% rabbit serum (Vector Laboratories, Burlingame, CA, USA) and 10% goat serum (Vector Laboratories) diluted in phosphate-buffered saline (PBS), and Avidin/Biotin Blocking Kit (Vector laboratories) were applied to the sections. Anti-digoxigenin antibodies (1:10000, Vector Laboratories Cat# MB-7000, RRID : AB_2336116) in PBS were applied to the slides for 30 min at room temperature. Biotinylated anti-goat antibody (1:500, Vector Laboratories Cat# BA-5000, RRID : AB_2336126) in PBS containing 5% rabbit serum was added to the sections for 10 min at room temperature. After this reaction, the sections were washed in PBS and incubated with VECTASTAIN Elite ABC Kit (Vector Laboratories). The slides were incubated with solution containing 3,3' -diaminobenzidine (Dako), nickel(II) ammonium sulfate, hexahydrate (12mg/mL, Nacalai Tesque Inc., Kyoto, Japan) and 0.01% H_2_O_2_. The reaction was stopped by PBS. The sections were mounted with Poly-Mount (Polysciences,Warrington, PA, USA) and analyzed using a microscope (BZ-X710, Keyence). In the case of Gram stain and ISH, Gram stain was performed after ISH color development.

### Comparisons of prostate microbiota between the BPE and non-BPE groups

To explore the association between the prostate microbiota and BPE, prostate tissue samples were collected and divided into the BPE and non-BPE groups, and the microbiota of the two groups were compared. The BPE group was defined as prostate volume ≥30 mL, and the non-BPE group as prostate volume <30 mL. Prostate volume was measured using magnetic resonance imaging or computed tomography by two urologists (KO and KT). The prostate volume was calculated *via* the anteroposterior (AP), craniocaudal (CC) and laterolateral (LL) diameters through the ellipsoid formula AP × CC × LL × 0.523. The prostate tissues were collected and submitted to bacterial DNA extraction and analysis of microbiota as described above. Alpha and beta diversity of the prostate microbiota in the BPE and non-BPE groups were compared in the same manner as above.LEfSe was also performed in the same manner as aboveto identify significantly different OTUs between the two groups.

### Ethical approval and consent to participate

The study protocol was reviewed and approved by the Institutional Review Board (IRB) of Osaka University Hospital (IRB no. 20350) and written informed consent was obtained from all patients.

## Results

### Comparisons of microbiota in prostate tissue and catheterized urine

**Figure 1:**
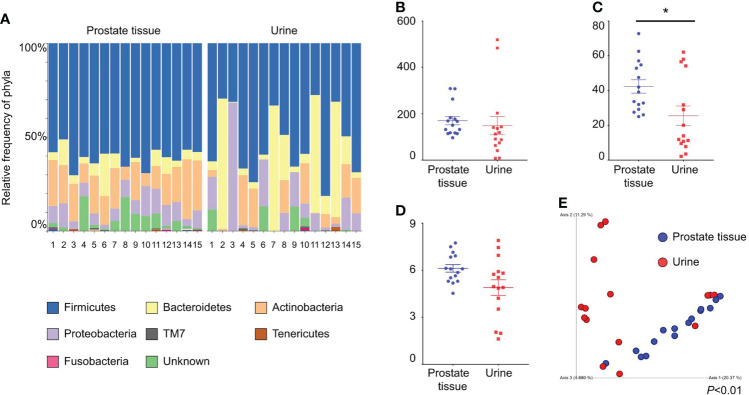
Comparison of alpha and beta diversity of the microbiota of prostate tissue and catheterized urine from 15 patients each. **(A)** Relative frequency of bacterial phyla in each sample. Each bar represents a subject sample, and each colored box represents a bacterial phylum. The height of a colored box represents the relative abundance of the bacterial phylum in the sample. **(B)** Chao1 index. **(C)** Faith’s PD index. **(D)** Shannon index. The date represents the mean and the SEM. **(E)** PCoA plots based on unweighted UniFrac distance. *P < 0.05.

We collected both prostate tissue and catheterized urine samples from 15 patients. The background characteristics of the patients are shown in **Table 1**. The relative frequencies of bacterial phyla in prostate tissue and catheterized urine are shown in **Figure 1A**. There was no difference in Chao1 index between the microbiota in prostate tissue and catheterized urine (mean ± standard SEM), 170 ± 17.9 vs 149 ± 39.3, *P*=0.67) (**Figure 1B**). The microbiota of prostate tissue had higher Faith PD index (42.3 ± 3.8 vs 25.5 ± 5.6, *P*=0.01) (**Figure 1C**) and tended to have higher Shannon index (6.1 ± 0.24 vs 4.9 ± 0.50, *P*=0.07) than urine had (**Figure 1D**). PCoA showed a significant difference between the microbiota of prostate tissue and catheterized urine (*P<*0.01) (**Figure 1E**). LEfSe showed that six OTUs had a significantly lower relative abundance and that 20 OTUs had a significantly higher relative abundance in the microbiota of prostate tissue than in that of urine (**Supplementary Figure 1**). These results demonstrated the difference in microbiota of prostate tissue and catheterized urine, and therefore suggested the presence of the prostate microbiota.

**Table 1:** Background characteristics of patients from whom prostate tissue and catheterized urine samples were taken.

		Value	range
No. of patients		15	
age(y)		74	(63-78)
prostate volume(mL)		47.7	(17.4-130)
Surgery	HoLEP	8	
	RALP	7	

Values are presented as the median (range) or number.

HoLEP, holmium laser enucleation of prostate; RALP, robot assisted laparoscopic prostatectomy.

### FISH for eubacteria in prostate tissue

**Figure 2:**
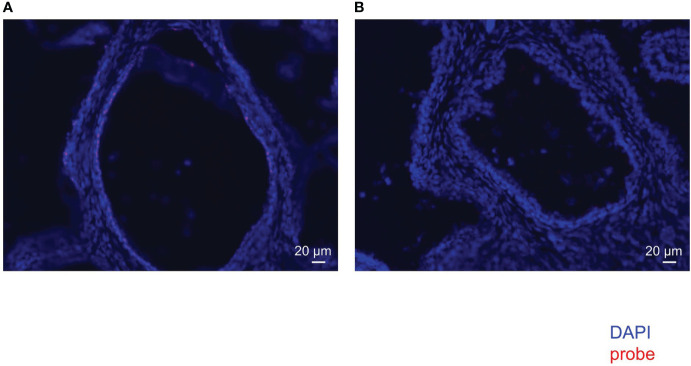
FISH with EUB338 probe (eubacteria specific) **(A)** and NON338 probe (negative probe for eubacteria) **(B)** in human prostate serial slices.

FISH of mouse intestine with EUB338 (**Supplementary Figure 2A**) showed emanated signals but FISH with NON338 (**Supplementary Figure 2B**) showed only autofluorescence. These results showed the specificity of the EUB338 probe for detecting eubacteria. FISH of the human prostate with EUB338 (**Figure 2A**) showed eubacterial signals but FISH with NON338 (**Figure 2B**) showed no eubacterial signal. These results demonstrated the presence of the prostate microbiota.

### ISH for eubacteria in prostate tissue

**Figure 3:**
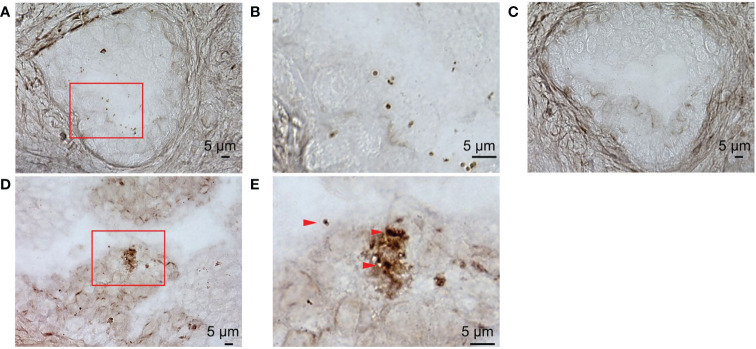
ISH with EUB338 probe (eubacteria specific) and NON338 (negative probe for eubacteria) on human prostate serial slices. **(A)** ISH with EUB338 probe. **(B)** Magnification of the red enclosed area in **(A, C)** ISH with NON338 probe. **(D)** Gram stain and ISH with EUB338 probe. **(E)** Magnification of the red enclosed area in **(D)** The Gram-stained and Ni-enhanced DAB colored cells are marked with arrowheads. ISH, *in situ* hybridization.

To examine detailed localization of the prostate microbiota under high magnification, we performed ISH for eubacteria. ISH of prostate tissue with EUB338 showed Ni-enhanced diaminobenzidine (DAB) color development in the prostatic duct (**Figure 3A**). High magnification detected 1 μm circular and elongated forms of color development which appeared to be cocci and rods (**Figure 3B**). No color development was observed by ISH with NON338 (**Figure 3C**). These results demonstrated that the prostate microbiota was localized in the prostatic duct. Gram stain and ISH with EUB338 also detected colored cells in the prostatic duct (**Figure 3D**). High magnification showed circular and elongated forms with Gram-stained cell walls and intracellular staining with Ni-enhanced DAB (**Figure 3E**). The ISH and Gram staining confirmed that Ni-enhanced DAB staining detected in the prostatic duct was definitely eubacteria.

### Comparisons of prostate microbiota between BPE and non-BPE groups

**Figure 4:**
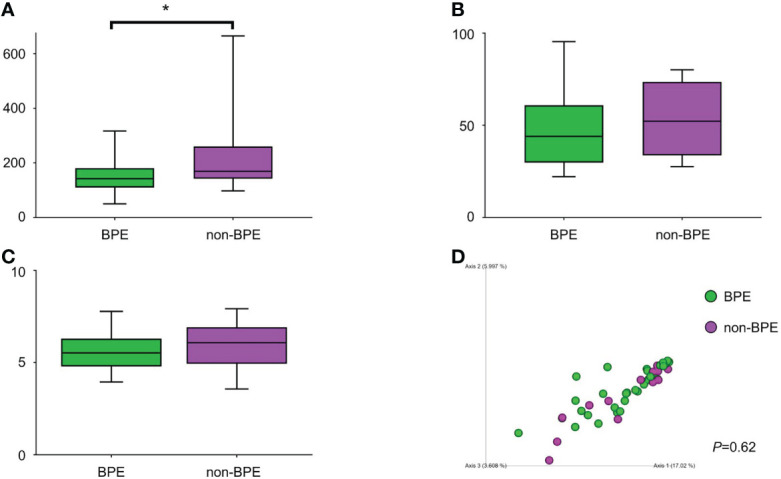
Comparison of alpha and beta diversity of prostate microbiota in the BPE and the non-BPE groups. **(A)** Chao1 index. **(B)** Faith’s PD index. **(C)** Shannon index. The data represent median (line in box) and interquartile range (box). **(D)** PCoA plots based on unweighted UniFrac distance showing the microbiota composition of prostate tissue in the PE and non-PE groups. *P < 0.05. BPE, benign prostate enlargement.

**Figure 5:**
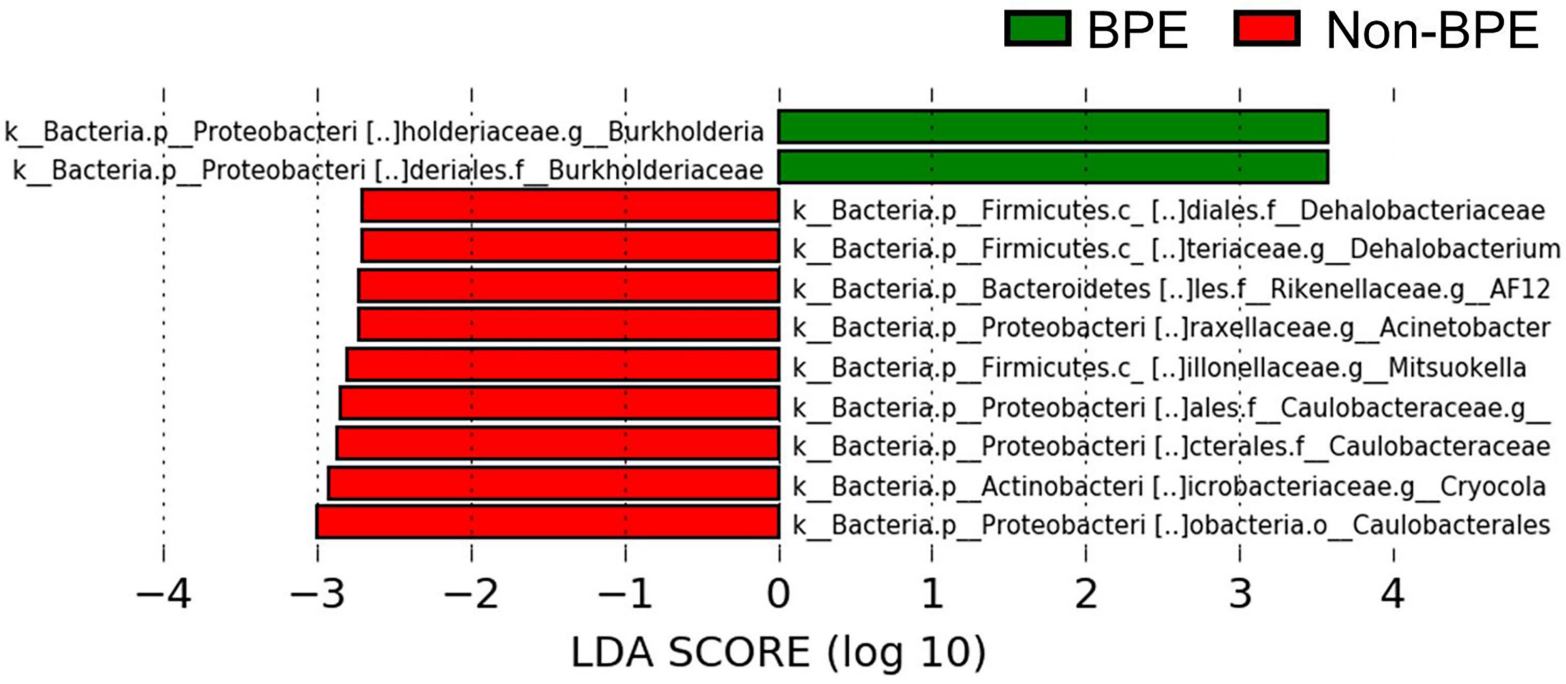
Comparison of the abundance of OTUs between the prostate microbiota of the BPE and the non-BPE groups (*P*<0.05 and LDA score > |2.0|). Green bars represent OTUs positively associated with BPE group and red bars represent OTUs positively associated with non-BPE group. LDA, linear discriminant analysis; BPE, benign prostate enlargement; OTUs, operational taxonomic units.

We compared the prostate microbiota between the BPE and non-BPE groups. Forty-nine prostate tissue samples were collected. Two FFPE specimens included prostate cancer and were excluded, leaving 47 tissue samples for analysis of their microbiota. The operations during which specimens were collected are shown in **Table 2**. There were 34 prostate tissue samples in the BPE group and 13 in the non-BPE group. The prostate microbiota of the BPE group had a lower Chao1 index (*P*=0.047) than that of the non-BPE group (**Figure 4A**). No significant difference was found in Faith’s PD index (**Figure 4B**) or Shannon index (**Figure 4C**). PCoA showed no significant difference between the two groups (*P*=0.62) (**Figure 4D**). LEfSe analysis showed that two OTUs had significantly higher relative abundance and nine OTUs had significantly lower abundance in the prostate microbiota of BPE group (**Figure 5**). Among the OTUs with significant differences between the prostate microbiota of the BPE and non-BPE groups, Burkholderia was the most abundantly detected OTU in both BPE and non-BPE groups (**Table 3**). As the abundance of Burkholderia and Burkholderiaceae is the same, Burkholderiaceae is all Burkholderia.

**Table 2:** Background characteristics of patients from whom prostate tissue was taken .

		BPE (n=34)		Non-BPE (n=13)	
Patient age(y)		71.5 (48-81)		72 (62-76)	
Prostate volume(mL)		46.4 (30.5-183)		26.8 (11.1-29.7)	
Surgery	HoLEP	9		2	
	RALP	17		9	
	Subcapsular prostatectomy	1		0	
	RALC	4		1	
	pelvic exenteration	3		1	

Values are presented as median (range) or number.

BPE, benign prostate enlargement; HoLEP, holmium lase enucleation of prostate; RALP, robot assisted laparoscopic prostatectomy; RALC, robot assisted laparoscopic cystectomy.

**Table 3:** The abundance of OTUs with significant differences between the prostate microbiota of the BPE and non-BPE groups.

OTUs	BPE		non-BPE	
g_Burkholderia	0.95% (0%-8.7%)		0.10% (0%-7.4%)	
f_Burkholderiaceae	0.95% (0%-8.7%)		0.10% (0%-7.4%)	
f_Dehalobacteriaceae	0% (0%-0.7%)		0% (0%-1.2%)	
g_Dehalobacterium	0% (0%-0.7%)		0% (0%-1.2%)	
g_AF12	0% (0%-0.7%)		0% (0%-0.7%)	
g_Acinetobacter	0% (0%-2.6%)		0% (0%-1.0%)	
g_Mitsuokella	0% (0%-0%)		0% (0%-1.4%)	
g_	0% (0%-0%)		0% (0%-0.45%)	
f_Caulobacteraceae	0% (0%-0%)		0% (0%-0.45%)	
g_Cryocola	0% (0%-1.6%)		0.04% (0%-1.3%)	
o_Caulobacterales	0% (0%-0%)		0% (0%-0.45%)	

Values are presented as median (range).

BPE, benign prostate enlargement; OTUs, operational taxonomic units.

## Discussion

We demonstrated the presence of the prostate microbiota by genetic analysis and histological studies. We also showed that the prostate microbiota was associated with BPE.

We analyzed the microbiota of 47 prostate tissues, 26 catheterized urine samples, and three saline samples as negative controls (**Supplementary Table 1**; **Supplementary Figure 3**). Firmicutes, Bacteroidetes, Actinobacteria and Proteobacteria accounted for >70% of all prostate tissue specimens, and in >80% of catheterized urine specimens, which is similar to previous studies (Jain et al., 2020; Hrbacek et al., 2021). Our study showed that the microbiota in prostate tissue had more phylogenetic diversity than the microbiota in catheterized urine according to Faith’s PD index. The difference in beta diversity showed that the bacterial composition in prostate tissue differed significantly from that in catheterized urine. These results demonstrated that the microbiota of prostate tissue was different from that of catheterized urine, and therefore, revealed the presence of prostate microbiota. When analyzing the microbiota of low-biomass specimens, contaminating DNA in DNA extraction kits and other laboratory reagents can impact the results (Salter et al., 2014). To minimize the effect of contamination, we performed 16S metagenomic analysis in a single sequence including sterilized saline samples as negative controls. In the analysis of 47 prostate tissues, 26 catheterized urine samples and three saline samples, the alpha and beta diversities of prostate tissue differed from those of catheterized urine and saline (**Supplementary Figure 4A–D**). These results are similar to the comparisons between microbiota of prostate tissue and catheterized urine from the same patient (**Figures 1B–E**) and therefore support the presence of the prostate microbiota.

We identified the presence of the prostate microbiota not only by 16S metagenomic analysis but also by FISH and ISH. FISH showed that bacteria were present in the prostate. ISH allowed more detailed observations and revealed that bacteria were located in the prostatic duct. We suppose that the bacteria in the prostate came either hematogenously or retrogradely through the urinary tract. The localization of bacteria in the prostatic duct suggests that they arrived in the prostate *via* the urinary tract rather than *via* blood vessels.

For clarification of the relationship between prostate microbiota and BPE, the prostate microbiota of the BPE group had a lower Chao1 index, which is used to measure species richness, than that of the non-BPE group. The urinary microbiota of the BPE group was not significantly different from that of the non-BPE group (**Supplementary Table 2**, **Supplementary Figure 5A–D**). Reduced diversity is considered to be one of the abnormal conditions of the microbiota, called dysbiosis (Petersen and Round, 2014). The gut microbiota has various effects on the host, and some host–microbe interactions are caused by more than one bacterial species (Faith et al., 2014). This suggests that more complex and diverse microbiota may have more health benefits for the host, and reduced diversity of microbiota is included in dysbiosis (Petersen and Round, 2014). Reduced diversity of microbiota has been reported in the associations between gut microbiota and inflammatory bowel disease (Santana et al., 2022), skin microbiota and atopic dermatitis (Clausen et al., 2016), and respiratory microbiota and asthma (Dickson et al., 2016). We hypothesized that the mechanism by which the reduced diversity of prostate microbiota is associated with BPE is inflammation, as inflammation is reported to have a correlation with prostate volume (Nickel et al., 2008). We performed Giemsa staining and ISH to examine leukocyte accumulation around the bacteria in the prostate. We also performed HE staining to examine if there was disruption of the prostatic duct. Giemsa stain and ISH showed no accumulation of leukocytes around the bacteria in the prostatic duct (**Supplementary Figure 6A**), and HE showed no disruption of the prostatic duct (**Supplementary Figure 6B**). Therefore, the presence of bacteria in the prostate does not necessarily cause obvious inflammation. Reduced diversity of prostate microbiota may be related to BPE by mechanisms other than inflammation, as bacteria in prostate tissue do not necessarily cause an inflammatory response. LEfSe analysis indicated higher abundance of Burkholderia in the BPE group. It is reported that Burkholderia cenocepacia, which is one of the species of the Burkholderia genus, adhere to epithelial cells in respiratory tract and promote IL-8 production and NF- κB activation (Saldías and Valvano, 2009). Burkholderia may also stimulate IL-8 production and NF- κB activation in prostate and promote proliferation of prostate stromal cells (Lucia and Lambert, 2008; Jang et al., 2021). Further studies are needed to identify and elucidate the causal relationship between Burkholderia and BPE.

There were some limitations to our study. First, the study is based on the data of Japanese. Prostate microbiota is expected to vary with genetic and environmental factors because gut and urinary microbiota vary with genetic and environmental factors (Adebayo et al., 2020). Second, there were multiple types of diseases in this study, and it is possible that prostate microbiota differs depending on the disease. Among the prostate tissue samples collected, 12 were from noncancer surgery and 35 were from cancer surgery. It has been reported that the microbiota differs between cancerous and noncancerous sites in cases of prostate cancer (Cavarretta et al., 2017), and we excluded cases in which prostatic cancer was found in the tissue adjacent to the tissue submitted to 16S metagenomic analysis. When the prostate microbiota was compared between noncancer and cancer surgery in this study, there was no significant difference in either alpha or beta diversity (**Supplementary Figure 7A–D**). However, we cannot deny the possibility that the prostate microbiota in noncancerous sites differed from the prostate microbiota when those patients did not have cancer. Third, the patients underwent different types of surgery. HoLEP was performed on 11 patients and the other operations on 36. Saline infusion in the urinary tract during HoLEP may affect the prostate microbiota. There were no significant differences in alpha or beta diversity between samples from HoLEP and those from the other surgeries (**Supplementary Figures 8A–D**). In a 16S metagenomic analysis of prostate microbiota, saline infusion during HoLEP appeared to have little effect.

## Conclusions

In this genetic and histological study of prostate microbiota, we demonstrated the presence and localization of prostate microbiota and the association between reduced diversity of prostate microbiota and BPE.

## Data availability statement

The datasets presented in this study can be found in online repositories. The names of the repository/repositories and accession number(s) can be found below: DNA Databank of Japan (DDBJ) Sequence Read Archive (DRA) under the accession number DRA014901.

## Ethics statement

The studies involving human participants were reviewed and approved by The institutional Review Board of Osaka University Hospital. The patients/participants provided their written informed consent to participate in this study.

## Author contributions

KO, KT, KF, HK, YK, DM, and SN conceived and designed research. KO, KT, GT, TI, SK, SF, and HK collected samples and clinical data. KO, KT, NU, SF, and HK performed research. KO, KT, HK, DM, SN, and YK analyzed data. KO, KT, DM, SN, YK, SS, and NN wrote the paper. All authors contributed to the article and approved the submitted version.
