# Supplementary figures and images for: Localization and potential role of prostate microbiota
Source: Front Cell Infect Microbiol. 2022 Dec 7;12:1048319. doi: 10.3389/fcimb.2022.1048319 (PMC9768196; doi:10.3389/fcimb.2022.1048319)

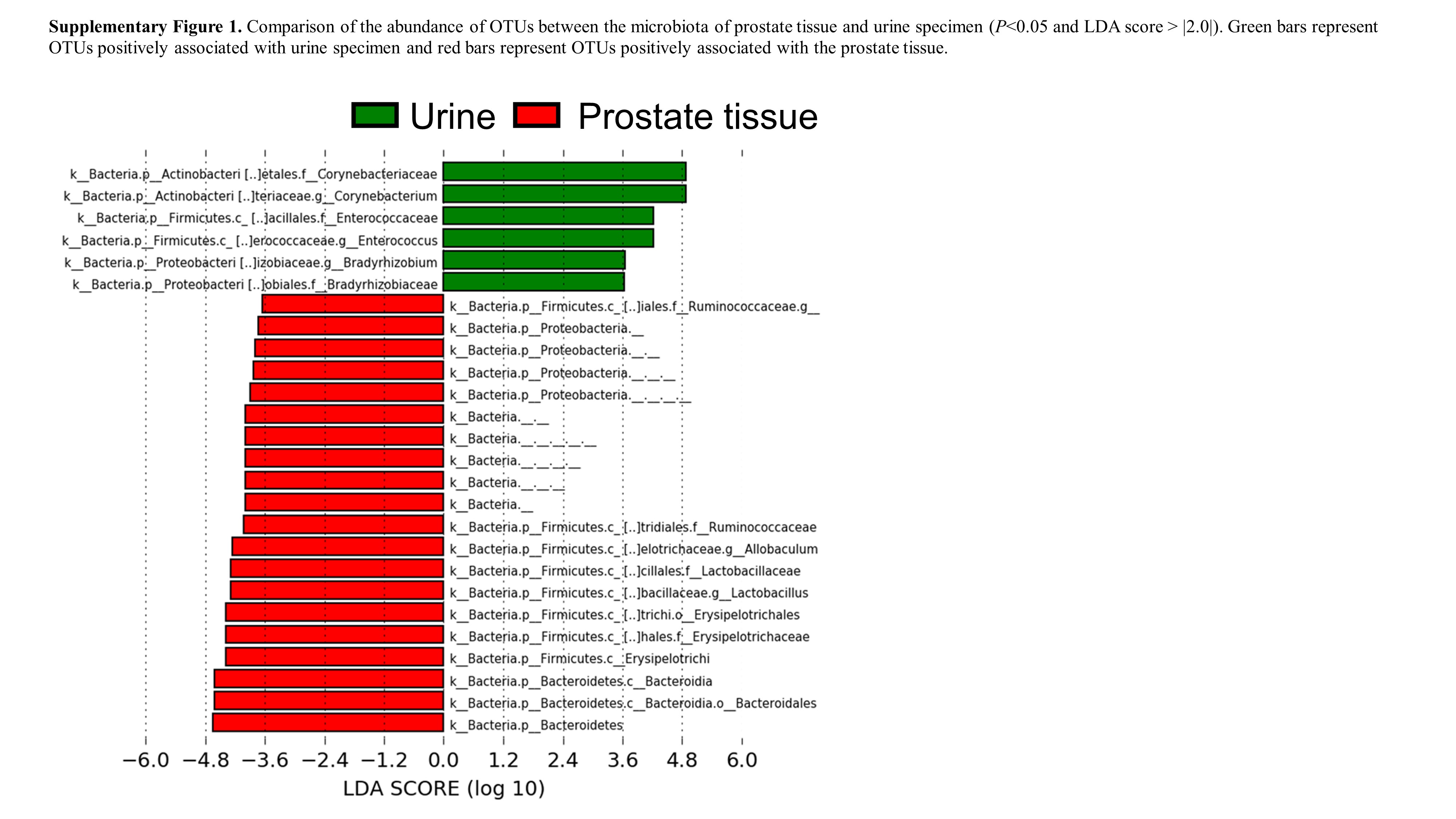

Supplement: Supplementary file 1 [file Image_1.jpeg]

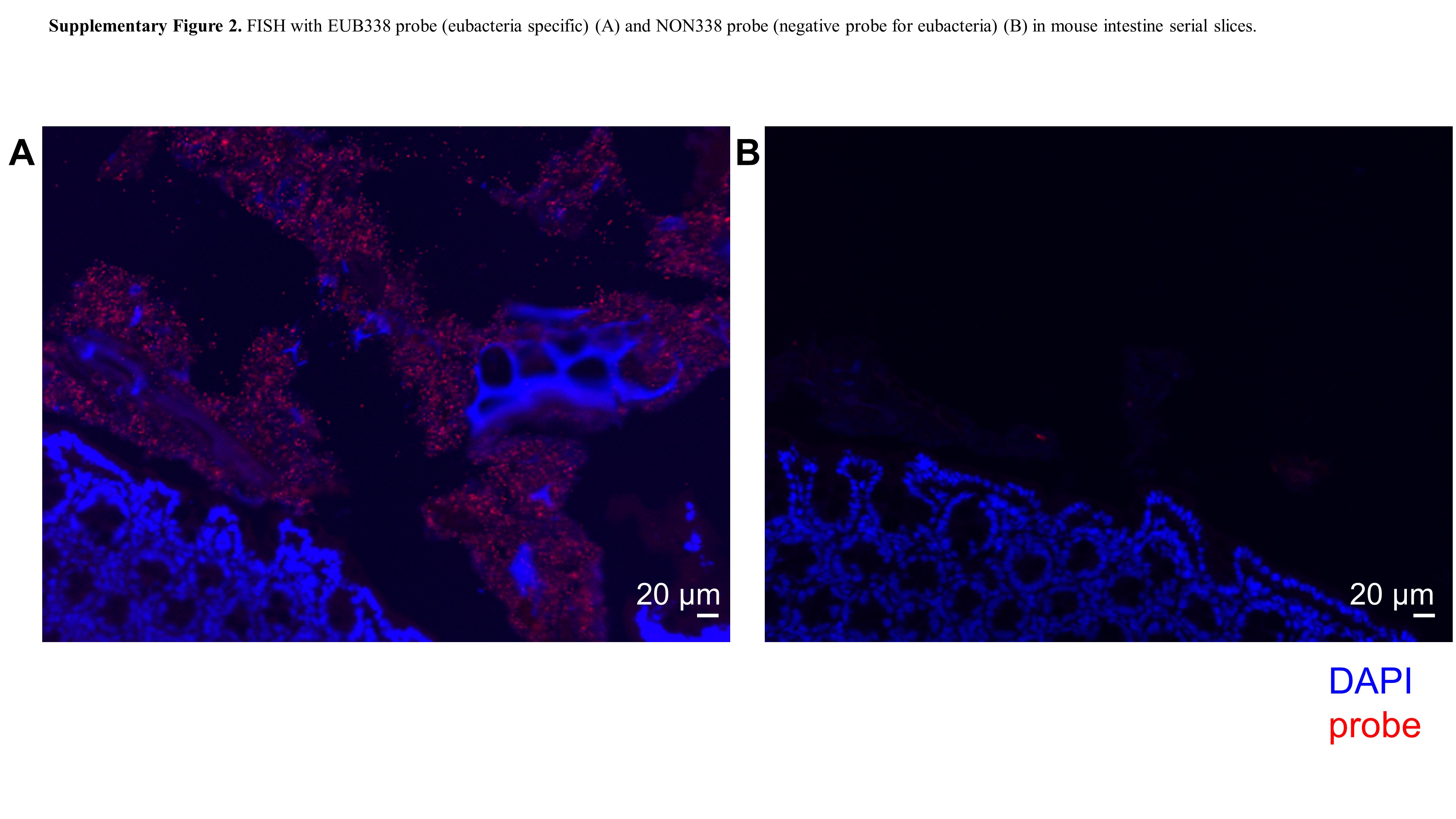

Supplement: Supplementary file 2 [file Image_2.jpeg]

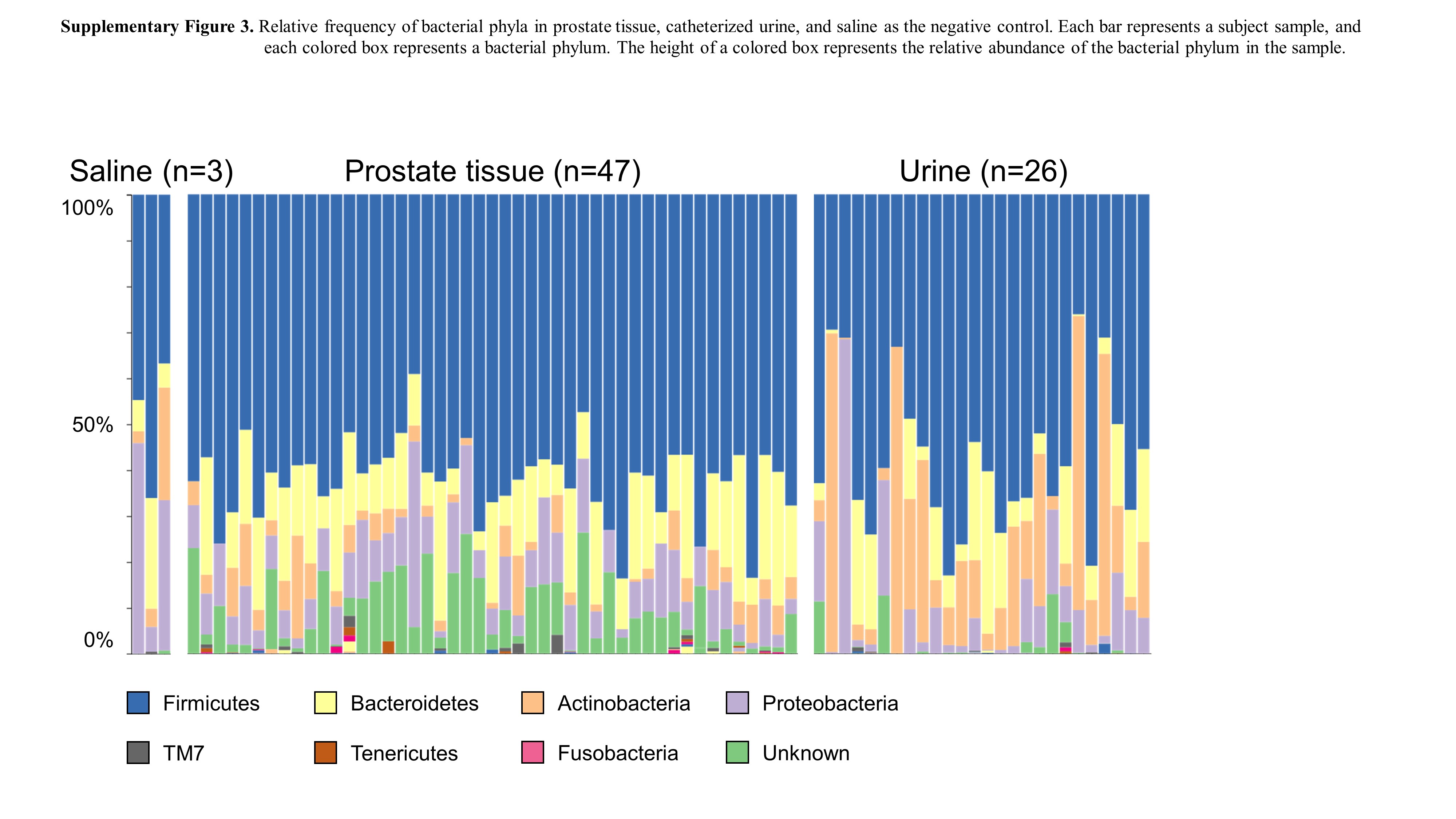

Supplement: Supplementary file 3 [file Image_3.jpeg]

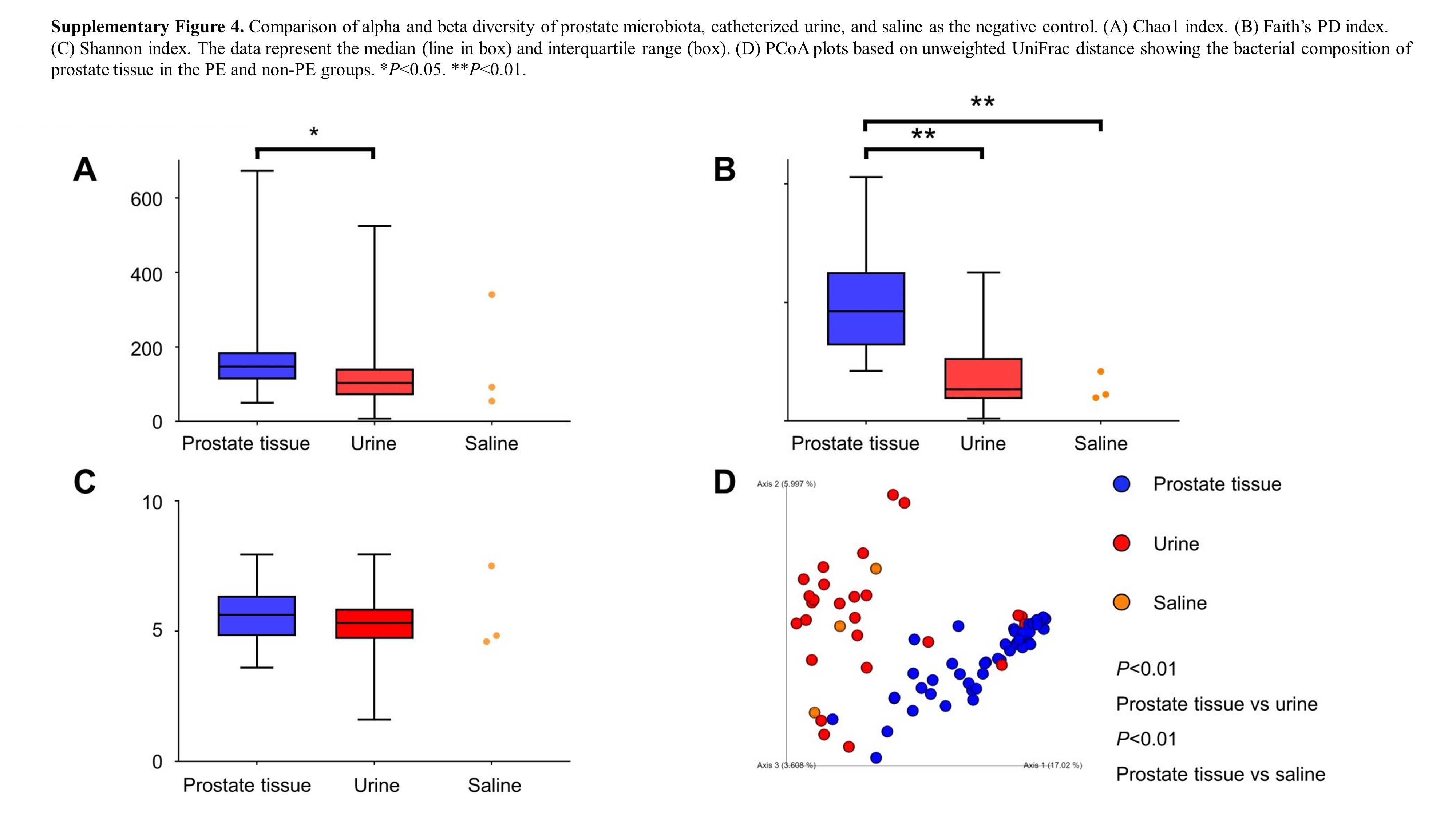

Supplement: Supplementary file 4 [file Image_4.jpeg]

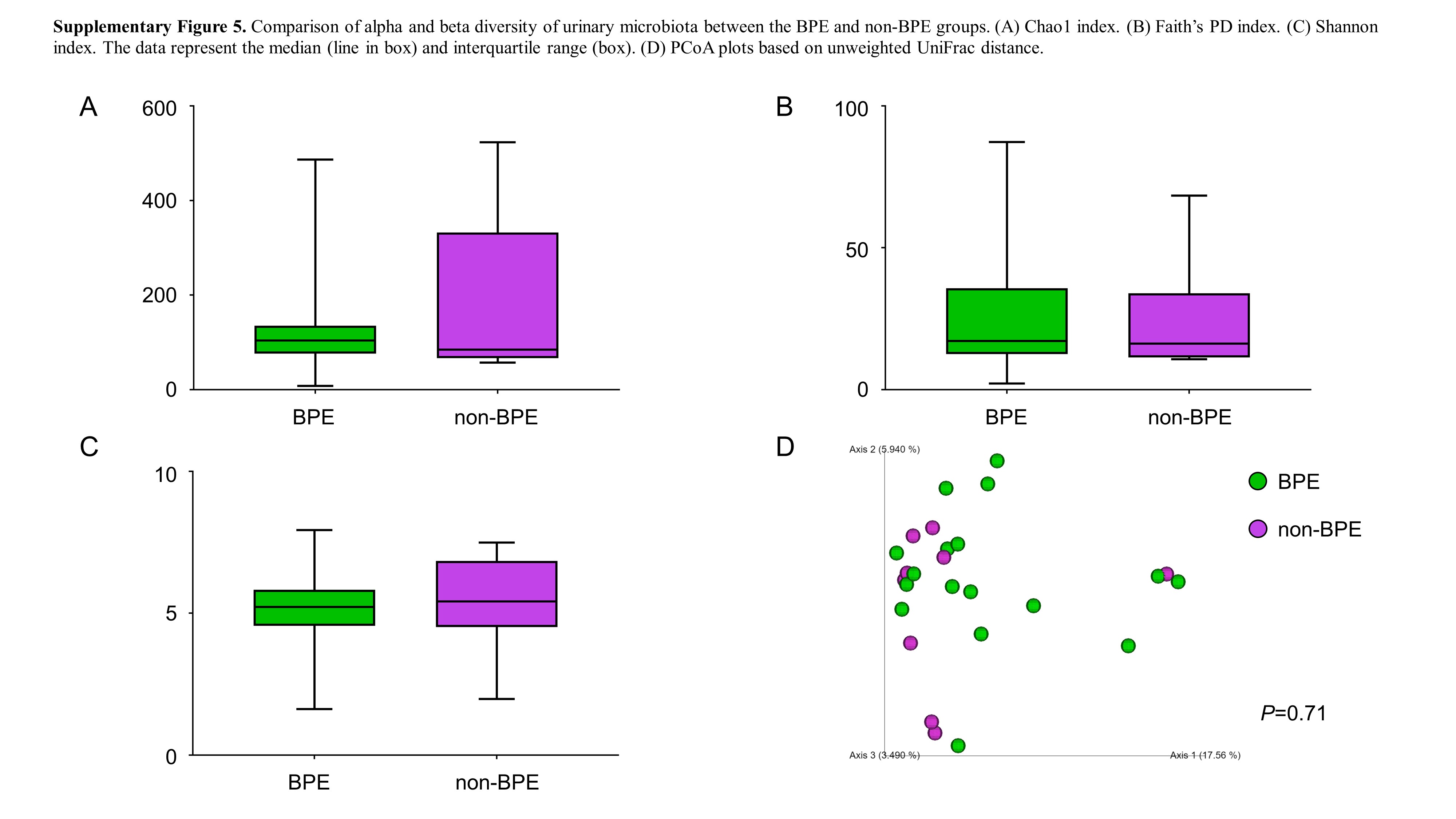

Supplement: Supplementary file 5 [file Image_5.jpeg]

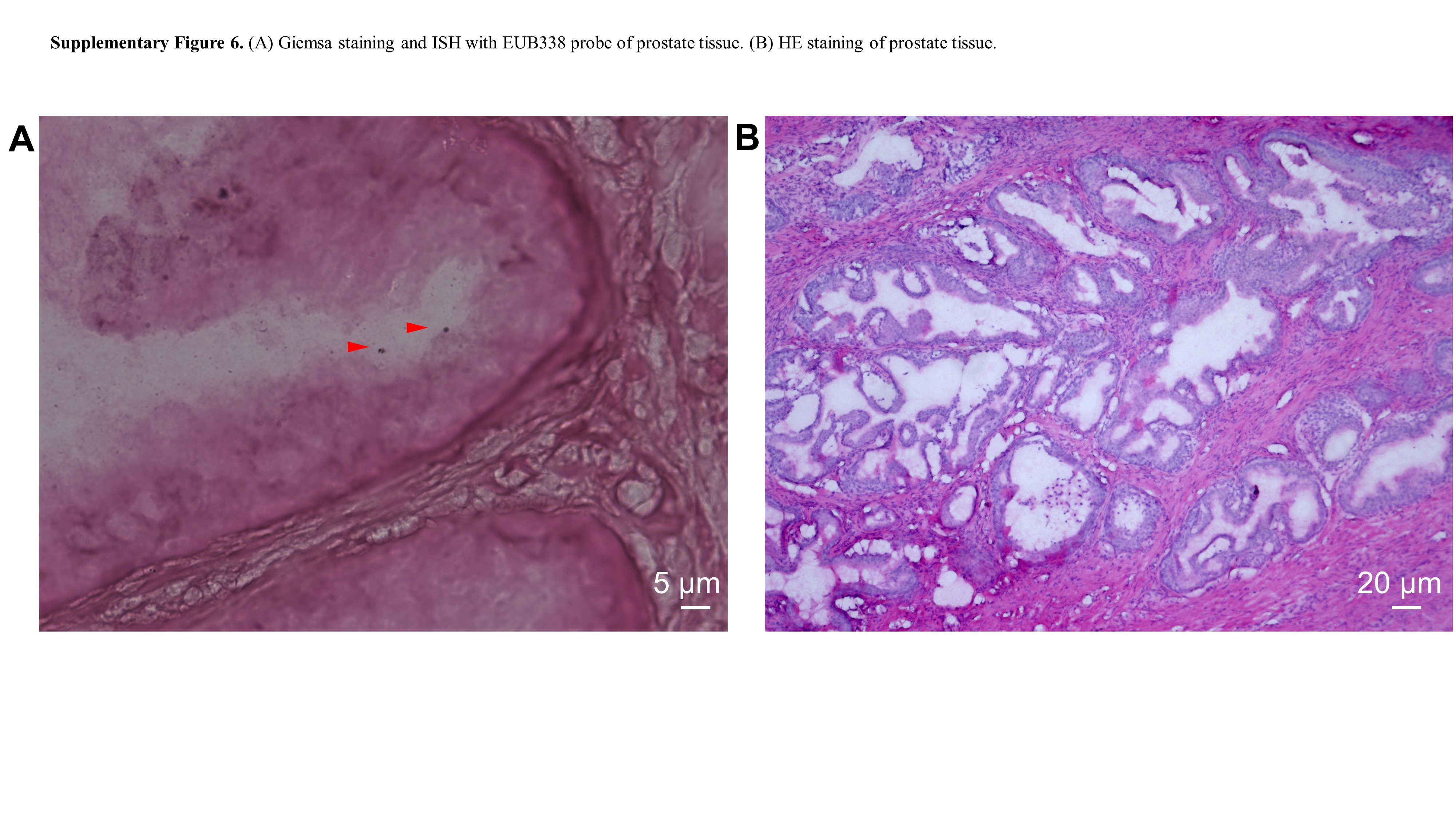

Supplement: Supplementary file 6 [file Image_6.jpeg]

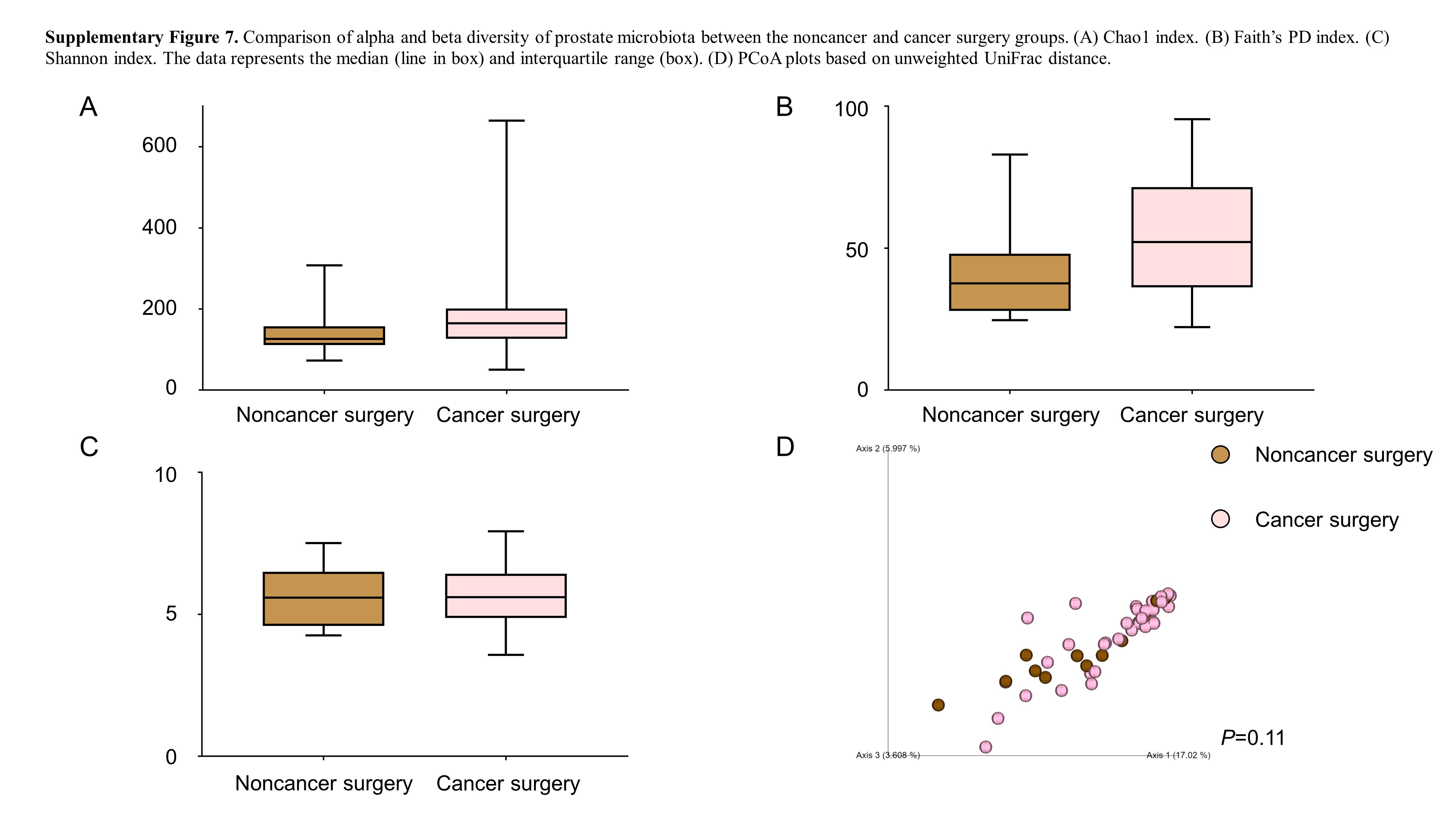

Supplement: Supplementary file 7 [file Image_7.jpeg]

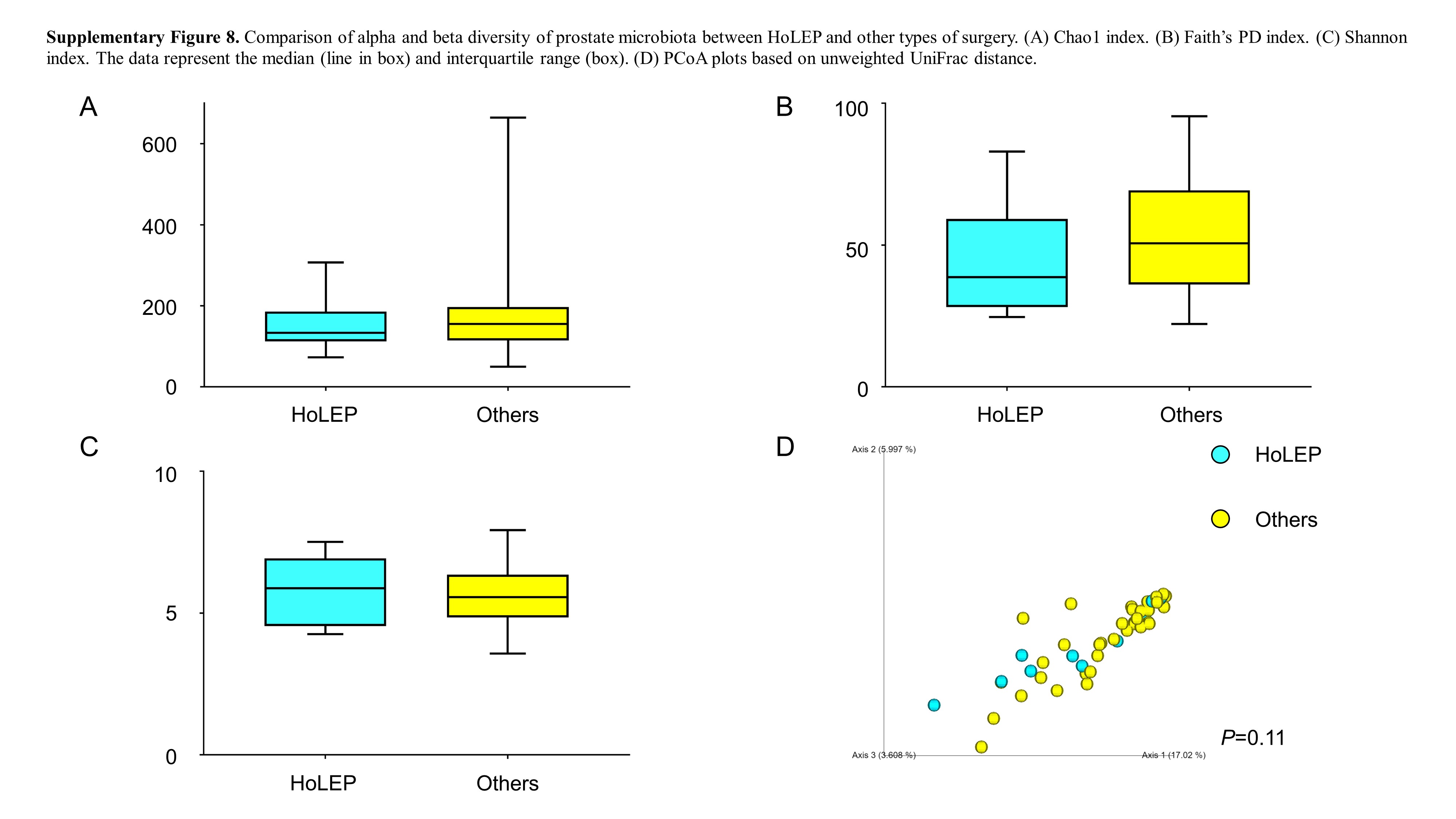

Supplement: Supplementary file 8 [file Image_8.jpeg]
