# Supplementary material for: Localization and potential role of prostate microbiota
Source: Front Cell Infect Microbiol. 2022 Dec 7;12:1048319. doi: 10.3389/fcimb.2022.1048319 (PMC9768196; doi:10.3389/fcimb.2022.1048319)
Supplement: Supplementary file 9 [file Table_1.docx]

**TABLE S1** Background characteristics of patients with 47 prostate tissue and 26 catheterized urine samples

|  |  | **Prostate tissue (n=47)** | | **Catheterized urine (n=26)** | |
| --- | --- | --- | --- | --- | --- |
| age(y) | | 71.5 | (48-81) | 69.5 | (26-78) |
| prostate volume(mL) | | 46.4 | (30.5-183) | 39.8 | (15.2-130) |
| Surgery | HoLEP | 9 |  | 8 |  |
|  | RALP | 17 |  | 10 |  |
|  | Subcapsular prostatectomy | 1 |  | 0 |  |
|  | RALC | 4 |  | 0 |  |
|  | pelvic exenteration | 3 |  | 0 |  |
|  | Donor Nx | 0 |  | 2 |  |
|  | LNx/RAPN | 0 |  | 6 |  |
| Values are presented as median (range) or number | | | | | |
| RALP, robot-assisted laparoscopic prostatectomy. RALC, robot-assisted laparoscopic cystectomy. Nx, nephrectomy. LNx, laparoscopic nephrectomy. RAPN, robot-assisted laparoscopic partial nephrectomy | | | | | |

**TABLE S2** Background characteristics of patients from whom catheterized urine samples were taken

|  |  | **BPE (n=17)** | | **Non-BPE (n=9)** | |
| --- | --- | --- | --- | --- | --- |
| Patient age(y) | | 62 | (26-76) | 70 | (42-78) |
| Prostate volume(mL) | | 47.7 | (34.5-130) | 25.1 | (15.2-29.7) |
| Surgery | HoLEP | 6 |  | 2 |  |
|  | RALP | 8 |  | 2 |  |
|  | Donor Nx | 1 |  | 1 |  |
|  | LNx/RAPN | 2 |  | 4 |  |
| Values are presented as median (range) or number | | | | | |
| RALP, robot-assisted laparoscopic prostatectomy. Nx, nephrectomy. LNx, laparoscopic nephrectomy. RAPN, robot-assisted laparoscopic partial nephrectomy. | | | | | |
